# Principles

**Published:** 1895-09

**Authors:** Frank S. Davis

**Affiliations:** Quincy, Mass.


### SOCIETY OF HOMŒOPATHICIANS.

### Meeting of 1895.

---

### First Day. Morning Session.

---

✓

### PRINCIPLES.

Frank S. Davis, M. D., Quincy, Mass.

The whole of anything is greater than any part of it. The union of its parts into a perfect *whole* constitutes the integrity of that body.

These axioms are applicable to this society of physicians. We join ourselves together under certain articles of government to which each member subscribes, and the integrity of our society will depend upon the soundness of the governing principles and upon the strict observance of them by each individual member of it.

Therefore, we expect every member to consider himself bound by the obligations taken, and to expect censure to follow if he transgress.

We cannot permit transgression to pass unnoticed without endangering our very existence.

It must not be forgotten that we are here for a purpose, the accomplishment of which is of far greater importance than is the pleasure or profit that may come to any portion of our membership.

Rather than be liable to a loss of principle we will submit to a loss of membership, because one or more unsound parts must make an unsound whole.

If our principles, rules, or Constitution and By-Laws are not perfect we can endeavor to perfect them; but after they have been adopted let them be tested by strict observance.

I ask no favor, I shall expect equal rights, and my obligations must be equal to those of every other member.

Let our requirements for membership be kept at the highest standard and rigidly lived up to. It will be much easier to *reject* than it will be pleasant to *regret* admission of any one. I believe we should observe the utmost care in every step taken to increase our membership, for if one member proves unfaithful the whole body suffers.

We believe in the immutability of natural laws.

We believe that Hahnemann discovered a natural law when he turned the search light of his master mind into the darkness which for centuries obscured the vision of every investigator in the realm of medicine.

We believe he has given the best and clearest definition of disease.

We believe he has given us the best method of ascertaining the healing power of medicines.

We believe he has given the best directions for the cure of disease by medicine.

We believe he has discovered the truth upon which rests the curative relation of the medicine to the disturbed life-force.

We believe he has given the best guide to the selection of the remedy.

We bind ourselves to follow his instructions in our practice, and not to proceed contrary to them.

Hahnemann describes *disease* as a disturbance of the spiritual life-force, manifested by symptoms subjective and objective. He says, § 16 of *The Organon*, our vital force, that spiritual dynamis cannot be reached nor affected except by a spiritual (dynamic) process, resulting from the hurtful influences of hostile agencies from the outer world acting upon the healthy organism and disturbing the harmonious process of life. Neither can the physician free the vital force from any of these morbid disturbances—*i. e.*, diseases—except likewise by spiritual (dynamic, virtual) alterative power of the appropriate remedies acting upon our spiritual vital force—through the omnipresent susceptibility of the nerves of the organism. Thus healing remedies can and actually do restore health and vital harmony only by virtue of their dynamic action upon the vital force, after those changes in the health of the patient (totality of symptoms) perceived by our senses have represented the disease to the attentively observing physician as completely as possible for the purpose of its cure.

Section 18. It is then unquestionably true that besides the totality of symptoms it is impossible to discover any other manifestations by which diseases could express their need of relief. Hence it undeniably follows that the totality of symptoms observed in each individual case of disease can be the *only indication* to guide us in the selection of a remedy.

Section 19. Now since *diseases* are definable only as *aberrations from the state of health*, which disclose themselves by symptoms, and since a cure also becomes possible only by *changing this aberration back into the healthy state*, we may readily understand how impossible it would be to cure disease by medicines unless these possessed the power of altering the state of health dependent on feelings and functions of the organism. In fact, the curative power of medicines must rest alone on their power of altering the *sensorial* condition of the body.

Section 20. * * * It is only through manifestations of their effect upon the state of health that this power of drugs is experienced and observed.

Section 25. But now actual experience, the only infallible oracle of medical art, teaches in every carefully conducted experiment that *that* drug proved in its effect upon healthy persons, to produce the greatest number of symptoms similar to those found in a case of disease to be cured, and when administered in properly potentized and diminished doses, will rapidly, thoroughly, and permanently cancel and turn into health the totality of symptoms of that disease. Experience also teaches that all drugs will unexceptionably cure diseases, the symptoms of which are as similar as possible to those of the drugs, and leave none uncured.

All the principles explained by Hahnemann were proved by him and his associates to be true, and to give the best results in practice.

His direction to use only *one* remedy at a time, and that the best results would follow its administration in a *potentized* form—is sound instruction and the result of experience—and must be accepted by all who desire the best success in the cure of all forms of sickness.

We have founded this society upon these principles, and trust no one will join us who is not prepared to defend them by a strict observance of all of them in practice. This important duty is imperative that the science of pure Homœopathy may endure.

Hahnemann has asked his followers to do exactly as he did, and, if we fail, to report our failures to the world.

How very few have done this!

And how large the number is who, although they have a good understanding of how he practiced, have lacked the courage to follow his instructions! There is much in evidence of the fact that the number is large, comprising very able physicians, who prefer to sacrifice principle in practice to popularity and a desire for riches or fame.

They adopt surgical methods, and various other means which act to suppress rather than to cure disease, although they must know the error of such practice.

Again, how many there are who have no information of the truths contained in *The Organon!* They hear of such practice, and ask where they can learn about this pure Homœopathy.

Is there not a growing need of united effort by those who do believe and practice Homœopathy to prevent this art of healing from becoming one of the *lost arts?* We think there is, and are willing to work so it may soon be impossible for any one to be able, truthfully, to say that they do not know of any one in the city of Boston who now practices pure Homœopathy.

It does not speak well for the instruction given at our medical schools, where a claim is made to a belief in Homœopathy, if in their vicinity it is not known beyond peradventure that some are true followers of Hahnemann's instructions.

We have formed this society for work, determined to exert ourselves so that the light we have received may not grow dim. And we hope that all who are in earnest and willing to be faithful will come in and help to carry aloft the principles of pure Homœopathy where they will not be pulled down and trampled in the dust.

We do not come here expecting to be protected in any unhomœopathic practice.

We do not invite others to come here for any such protection.

We hope that here may congregate the faithful physicians, and that they will work together to the end that each may help and be helped to a better understanding and a higher appreciation of the principles of scientific medicine which Hahnemann elaborated and explained so fully in *The Organon*.

These principles have never been proved false, and upon them we rest our faith. They are our guide in practice. They give us courage to promise help to the sick, when without them we could find no relief at hand.

With the acceptance of these principles as our guide, there should come the responsibility of doing as Hahnemann did—work continually for their promulgation throughout the breadth of our country, and cease not to practice them on every occasion where our services to the sick are required.

